# Management of pregnancy complicated by ankylosing spondylitis: A case report and literature review

**DOI:** 10.1002/ccr3.2085

**Published:** 2019-03-06

**Authors:** Asumi Midorikawa, Liangcheng Wang, Tomoyuki Kuwata, Yoshie Taniguchi, Isao Horiuchi, Chikako Matsushita, Kenro Chikazawa, Kenjiro Takagi

**Affiliations:** ^1^ Perinatal and Maternal Center of Saitama Medical Center Jichi Medical University Saitama Japan; ^2^ Department of Anesthesia Saitama Medical Center, Jichi Medical University Saitama Japan

**Keywords:** ankylosing spondylitis, cesarean section, obstetrics anesthesia, pregnancy, vaginal delivery

## Abstract

Pregnancy complicated by ankylosing spondylitis is rare. Labor assistance and instrumental delivery may be difficult due to hip stiffness. Restriction in lumbar flexion may cause difficulties in administering neuraxial analgesia. Difficult intubation for general anesthesia due to limited neck mobility is another potential risk that must be considered.

## 1 INTRODUCTION

Ankylosing spondylitis (AS) is a chronic inflammatory rheumatic disease mainly characterized by pain and stiffness of the axial joints.^1^ The prevalence is approximately 7.4‐31.9 per 10 000 individuals^2^; disease onset is most frequently seen in the third or fourth decade of life.^3^ The cause of AS is unknown, although variations in the human leukocyte antigen B‐27 (HLA‐B27) are recognized to be strongly associated with the development of the disorder.^4^ The occurrence of AS is three times more frequent in males compared with females, and fertility is not considerably affected.^5^ However, the management of AS‐complicated pregnancy has rarely been reported in the literature. Herein, we present a case of a pregnant woman with AS and discuss the potential challenges in labor management.

## 2 CASE REPORT

**Figure 1:**
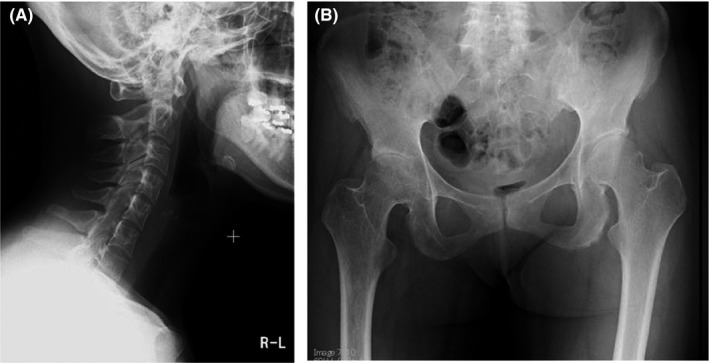
Radiograph of the patient at 33 y of age when ankylosing spondylitis was diagnosed. A, Radiograph of the neck. Osteosclerosis of the posterior surface of cervical vertebra was observed. B, Radiograph of both hips and the pelvis. Osteoarthritis of the right hip with joint space narrowing was observed

Written informed consent was obtained to report the case. A 34‐year‐old G2P1 pregnant woman diagnosed with AS presented at the obstetric outpatient clinic at 18 weeks of gestation. She experienced back pain when she was 25 years old; these symptoms made walking difficult during her first pregnancy at 31 years old. Following her first vaginal delivery, she was able to walk although pain persisted to a lesser degree than during pregnancy. Radiography revealed osteosclerosis of the posterior surface of cervical vertebrae and osteoarthritis of the right hip with joint space narrowing (Figure 1). The initial diagnosis of AS was made at 33 years of age.

Pain was managed by the administration of nonsteroidal anti‐inflammatory drugs (NSAIDs) and acetaminophen. After the present pregnancy was diagnosed, only acetaminophen continued to be administered, but pain increased during the present pregnancy. She complained of right hip pain, with a visual analog scale (VAS) score of 7/10. A dosage of 5 mg of oral prednisolone was administered daily from 18 weeks of gestation, and thereafter, the symptoms temporarily improved to 0/10 on the VAS. Unfortunately, the symptoms relapsed at 31 weeks of gestation. At 32 weeks of gestation, she complained of restricted neck mobility with difficulty gargling, restricted lumbar mobility that caused difficulties in bending her back, and restricted right hip joint mobility with a limitation of 10º of abduction, which also affected internal and external hip rotation. Peripartum management was discussed and planned by obstetricians and anesthesiologists. Given that only hip abduction and rotation, but not flexion, were limited, it was considered that an attempt of vaginal delivery would be possible when spontaneous labor occurred. However, if an emergency cesarean section (CS) was necessary during labor owing to other obstetric complications such as nonreassuring fetal status, special anesthesia management may be required because of the possible failure of spinal anesthesia due to calcified spinal ligaments and difficult tracheal intubation and airway management for general anesthesia. In such cases, the preparation of awake fiberoptic intubation or supraglottic airway device insertion should be considered.

At 38 weeks of gestation, the patient was admitted to our hospital because of membrane rupture. Labor analgesia was not provided. Oxytocin administration was required due to prolonged second stage of labor. Vacuum delivery with episiotomy left of the midline, which was opposite to the restricted right hip joint, was performed due to fetal bradycardia at birth. A healthy baby with a weight of 3358 g was successfully delivered. Both mother and neonate had a good postpartum course. Prednisolone and NSAIDs were started after pregnancy.

## 3 DISCUSSION

**Figure 2:**
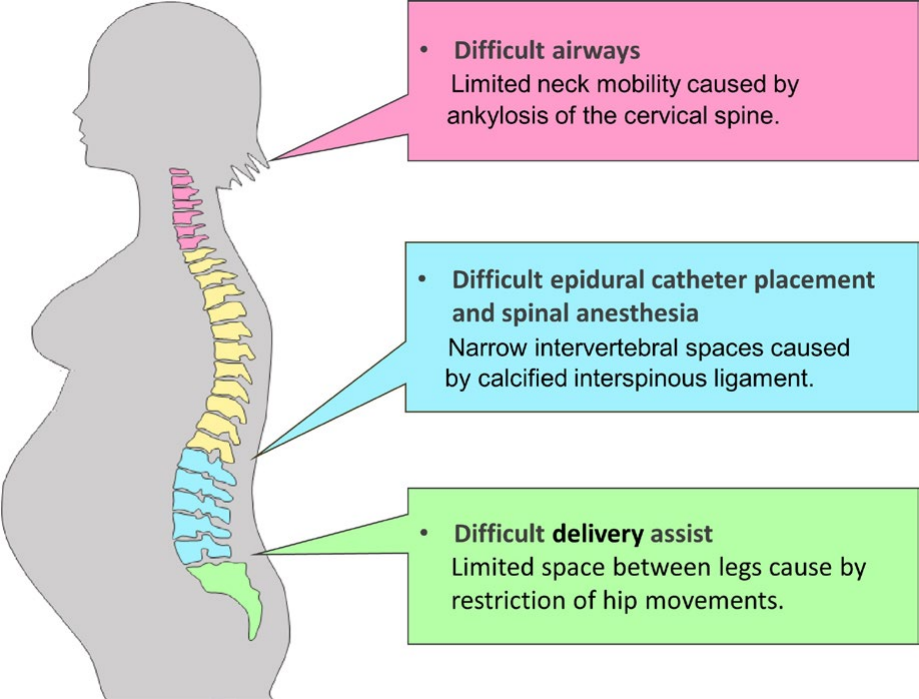
Major risk factors that require special assessment during labor in pregnant women with ankylosing spondylitis

Despite the feasibility of vaginal delivery in pregnant women with AS, three major risks in labor require special assessment: (a) difficult labor assistance and difficult instrumental delivery due to hip stiffness, (b) difficult neuraxial analgesia due to restriction of lumbar flexion and calcification of spinal ligaments when labor analgesia or emergency CS is required, and (c) difficult general anesthesia due to limited neck mobility and, therefore, airway access (Figure 2).

Theoretically, the restriction of hip joint mobility may be a mechanical hindrance that prevents the fetus from exiting the birth canal, increasing the risk of emergency CS. The rate of CS in pregnancies complicated by AS patients reported by Jakobsson et al and Zbinden et al was as high as 28.9‐39.7%^6^, ^7^ (Table 1). However, it appears that pre‐eclampsia or severe cases that required administration of tumor necrosis factor inhibitors, disease‐modifying antirheumatic drugs (DMARDs), or corticosteroids are the main causes of vaginal delivery failure in their studies, rather than hip stiffness. Similarly, the CS rate was 13.3%‐44.8% in patients with rheumatoid arthritis (RA) observed in other studies,^7^, ^8^ where the use of DMARD is the only factor associated with the need for CS (Table 2). Therefore, AS appears to have a minimal influence on the feasibility of vaginal delivery as far as hip joint disorders are concerned. Furthermore, Monaghan et al reported a case of bilateral total hip replacement with subsequent successful vaginal delivery of a baby weighing 3780 g with a bilateral abduction restriction <30º,^11^ indicating that the restriction of hip movement has no obvious influence on vaginal delivery. Other complications of pregnancies with AS such as preterm birth and small for gestational age have also been reported in the literature. However, the reported prevalence rates differ across studies (Tables 1 and 2), and the potential risk for the need for CS with these complications is unclear. Since there is a higher CS rate observed in severe cases,^6^ we believe that only severe cases of AS may require a higher priority for CS. In general, if AS is under control and other obstetric complications such as breech presentation, nuchal cord,^12^ or pre‐eclampsia are absent, limiting the delivery options is not essential. Conversely, it is possible that labor assistance may be affected due to limited space; an episiotomy performed opposite to the side of hip joint disorder may help reduce the risk of unexpected perineal laceration. Vacuum extraction may be a good option when an instrument‐assisted delivery is required during labor. It is unclear if forceps insertion will be restricted by hip stiffness. For this reason, we did not attempt to use forceps in the present case.

**Table 1:** Cesarean section rate of pregnancy complicated with ankylosing spondylitis/axial spondylarthrosis

Year	Author	Pregnancies	Mean maternal age	CS	CS rate (%)	Preterm birth rate (%)	SGA (%)	Risk factor for CS
2016	Jakobsson	388	32	112	28.9	9.0	3.1	pre‐eclampsia, administration of TNFi, DMARD, or corticosteroids
2018	Zbinden	78	32	31	39.7	11.4	11.4	

CS, cesarean section; DMARD, disease‐modifying antirheumatic drug; SGA, small for gestational age; TNFi: tumor necrosis factor inhibitor.

**Table 2:** Cesarean section rate of pregnancy complicated with rheumatoid arthritis/chronic inflammatory arthritis

Year	Author	Pregnancies	Mean maternal age	CS	CS rate (%)	Preterm birth rate (%)	SGA (%)	Risk factor for CS
2015	Bharti	440	32.7	182	41.6	17.7	9.0	
2018	Zbinden	86	32	43	44.8	18.2	16.2	DMARD use
2011	Wallenius	414^a^	NA	72	17.4	8.7	13.8	
		413^b^	NA	55	13.3	2.6	6.0	
2009	de Man	152	32.5	17	16.4	8.6	3.3	

CS, cesarean section; DMARD: disease‐modifying antirheumatic drug; NA, not available; SGA, small for gestational age.

a Nullipara.

b Multipara.

When labor pain control or CS is needed, challenges may remain. Stiffness of the axial joints and calcified spinal ligament may cause extreme difficulty in placing the epidural catheter or in inserting the spinal needle.^13^, ^14^ Narrow intervertebral spaces also make it difficult to perform epidural or spinal anesthesia. When regional anesthesia fails or an emergency CS is required, general anesthesia should be the first choice, but intubation may be difficult due to limited neck mobility. In addition, catastrophic neurological complications in cases with severe AS have been reported following emergency intubation in ICU.^15^ If difficult tracheal intubation is anticipated, awake fiberoptic intubation or supraglottic airway device insertion should be considered during induction of general anesthesia.

In conclusion, pregnancy complicated by AS is a rare condition. Despite the feasibility of vaginal delivery, difficulties in labor assistance, spinal anesthesia, and access to airways for general anesthesia are potential risks that need obstetricians’ and anesthesiologists’ attention.

## CONFLICT OF INTEREST

None declared.

## AUTHOR CONTRIBUTIONS

AM, LW, TK, and KC: wrote the manuscript. CM: involved in management of labor. IH and YT: involved in peripartum management. KT: supervised the project. All authors: discussed the results and contributed to the final manuscript.
